# Tenosynovitis caused by *Scedosporium apiospermum* infection misdiagnosed as an *Alternaria* species: a case report

**DOI:** 10.1186/s12879-016-2098-6

**Published:** 2017-01-14

**Authors:** Choon-Mee Kim, Sung-Chul Lim, Joa Kim, Hoe-Soo Jang, Jong-Hun Chung, Na-Ra Yun, Dong-Min Kim, Piyush Jha, Babita Jha, Seok Won Kim, Sook Jin Jang, Jong Hee Shin

**Affiliations:** 1 Premedical Science, College of Medicine, Chosun University, Gwangju, Republic of Korea; 2 Department of Pathology, School of Medicine, Chosun University, Gwangju, Republic of Korea; 3 Department of Internal Medicine, School of Medicine, Chosun University, Gwangju, Republic of Korea; 4 Department of Neurosurgery, School of Medicine, Chosun University, Gwangju, Republic of Korea; 5 Department of Laboratory Medicine, School of Medicine, Chosun University, Gwangju, Republic of Korea; 6 Department of Laboratory Medicine, Chonnam National University Medical School, Gwangju, Republic of Korea

**Keywords:** *Scedospermum apiospermum*, Tenosynovitis, Debridement, Case report

## Abstract

**Background:**

*Scedosporium apiospermum*, which can usually be isolated from soil, polluted stream water and decaying vegetation, is increasingly recognized as an opportunistic dematiaceous fungus. The mortality rate of infection in immunocompromised hosts is over 50%. *S. apiospermum* is commonly responsible for dermal and epidermal infections (i.e., mycetoma) after traumatic penetration.

**Case presentation:**

A 73-year-old woman was admitted to our hospital complaining of painful swelling and tenderness on the dorsum of the proximal left wrist and hand. The symptoms had persisted for approximately 2 months. A physical examination revealed a 4 x 3 cm, poorly defined, erythematous papule, which was fluctuant, with pustules and crusts on the dorsum of the left hand.

**Conclusions:**

We report a very rare case of tenosynovitis caused by *S. apiospermum* infection. We identified the infectious agent via molecular DNA sequencing. The infectious agent was initially misidentified as an *Alternaria* species by microscopic examination with lactophenol cotton blue (LPCB) staining. The infection was successfully treated with debridement and adjuvant fluconazole therapy.

## Background

*Scedosporium apiospermum* is an asexual anamorph of the fungus *Pseudallescheria boydii* and can usually be isolated from soil, polluted stream water and decaying vegetation [1]. This pathogen accounts for approximately 20% of all non-*Aspergillus* mold infections in organ transplant recipients [2]. *S. apiospermum* is increasingly recognized as an opportunistic fungus. The infection mortality rate is over 50% in immunocompromised hosts [3, 4]. Here, we report a case of tenosynovitis caused by an *S. apiospermum* infection that was initially misidentified as an *Alternaria* species. The infection was successfully treated with debridement and adjuvant fluconazole therapy.

## Case presentation

A 73-year-old woman was admitted to Chosun University Hospital, Gwang-ju, Korea. She was complaining of painful swelling and tenderness on the dorsum of the proximal left wrist and hand. The symptoms had persisted for approximately 2 months. A local clinic suspected cellulitis and administered cefoperazone/sulbactam for 10 days. However, the wound became worse, and erythema formed on the left hand. She had a clinical history of hypertension (20 years), diabetes (2 months), and a left distal radial and ulnar styloid process fracture that had occurred 2 years prior. In addition, she had undergone a discectomy at the L4-5 level one year previously.

A physical examination revealed a 4 × 3 cm, poorly defined, erythematous papule which was fluctuant, with pustules and crusts on the dorsum of left hand. Movements of the wrist, hand and fingers were slightly restricted and painful. Aspirated fluid from the lesion was obtained for mycological examination, and skin biopsies were obtained for histological and mycological examination.

**Fig. 1:**
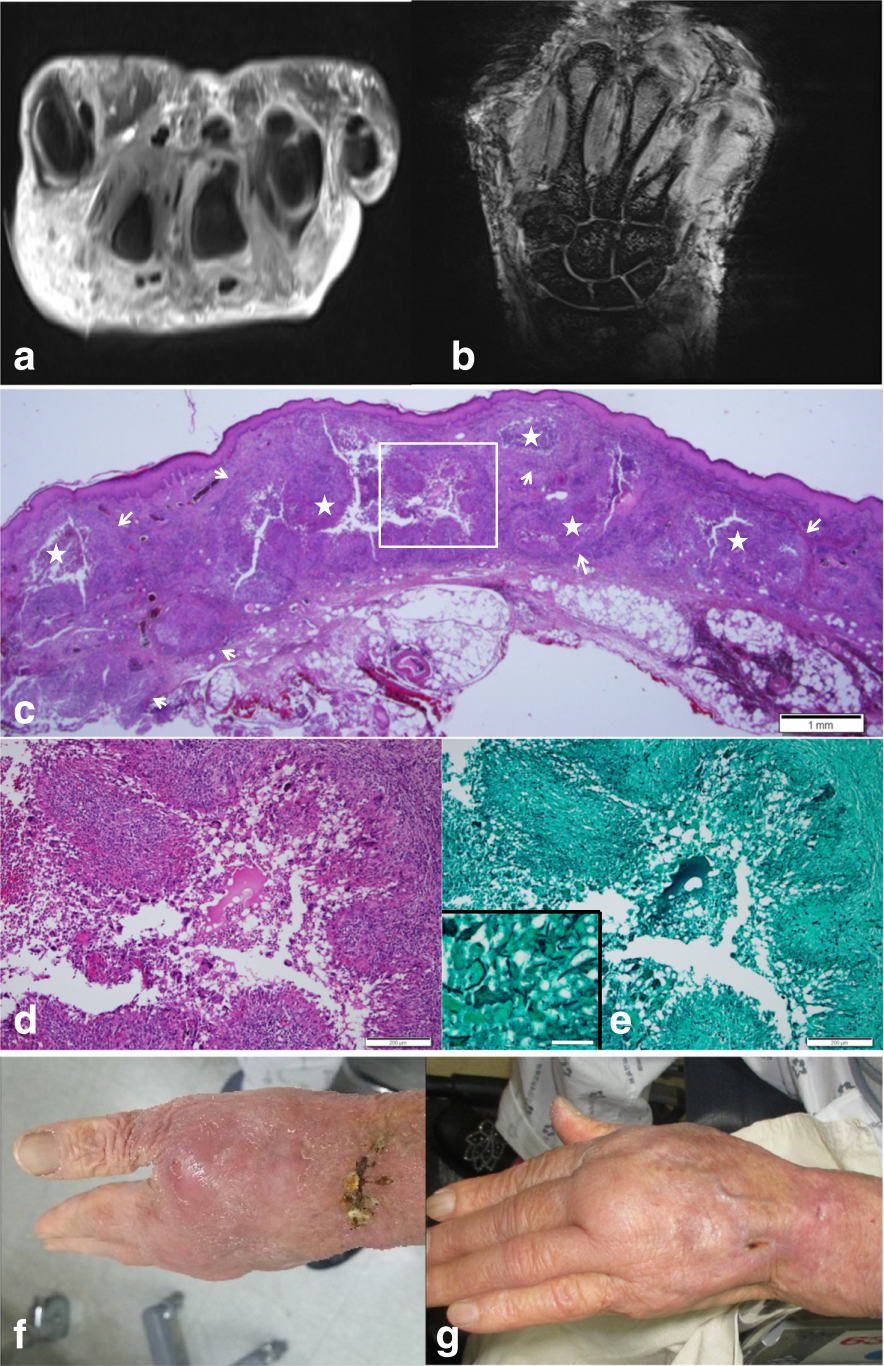
**a** MRI T2 weighted contrast transverse image reveals diffuse edematous swelling in the dorsum of the wrist, fluid in the extensor tendon sheath and fluid collection in the distal radio-ulnar joint. **b** MRI T1 weighted coronal image reveals diffuse paratendinous fluid in the extensor tendon sheath. **c** Skin biopsy shows multiple variable-sized granulomas (*arrows*) bearing central microabscesses (*asterisks*) in the dermis. H&E staining, Scale bar measures 1 mm. **d** Higher magnification of the image; a *boxed* area shows a well-formed palisading granuloma consisting of epithelioid cells, lymphocytes and multinucleated giant cells. H&E staining, Scale bar measures 200 μm. **e** Silver staining demonstrates septated fungal hyphae in the granuloma. Gomori methenamine silver staining, Scale bar measures 200 μm. Inset shows a higher magnification of the fungal hyphae. Scale bar measures 50 μm. **f** Skin photo on the dorsum of the *left* hand (before treatment). **g** Skin photo on the dorsum of the *left* hand (after treatment one month later)

The laboratory findings revealed that the white blood cell (WBC) count was 9360/mm^3^, the erythrocyte sedimentation rate (ESR) was 24 mm/h, and the C-reactive protein (C-RP) level was 1.13 mg/dL. An X-ray of the left hand revealed a healed fracture of the distal radius. Magnetic resonance imaging (MRI) of the left wrist revealed cellulitis of the dorsum of the wrist and hand and tenosynovitis of the extensor tendons (Fig. 1). Histological sections of the skin biopsies were stained with Gomori methenamine silver and revealed acute and chronic granulomatous inflammation with fungal hyphae and spores.

The clinical specimens were cultured on Sabouraud’s dextrose agar (SDA) containing chloramphenicol at 30 °C. On the 9th day, the culture yielded grey soft fungus on the front and dark-brown fungus on the other side. The clinical isolate was initially identified as an *Alternaria* species via a microscopic examination with lactophenol cotton blue (LPCB) staining. No other microorganisms were isolated from the clinical specimens. Because the wounds had improved, we continued treatment with wound irrigation, debridement and adjuvant fluconazole therapy with IV fluconazole 400 mg once a day for 14 days and changed to oral fluconazole 200 mg for 24 days. DNA sequencing of total genomic DNA extracted from purified colonies was performed to confirm the microbiological species.

Gene sequence analysis of the ITS region and D1/D2 regions of the 26S ribosomal DNA of rRNA genes was carried out. The ITS region (including the 5.8S rRNA gene) and 26S rRNA gene D1/D2 domains were amplified with the primer pair pITS-F (5′-GTCGTAACAAGGTTAACCTGCGG-3′) and pITS-R (5′-TCCTCCGCTTATTGATATGC-3′) and the primer pair NL1 (5'-GCATATCAATAAGCGGAGGAAAAG-3') and NL4 (5'-GGTCCGTGTTTCAAGACGG-3'), respectively [5]. The fungus was identified (www.ncbi.nlm.nih.gov/BLAST) as *Pseudallescheria boydii* (anamorph: *S. apiospermum*) with 100% homology according to the BLASTn program.

In vitro susceptibility testing of the mold isolate against amphotericin B, itraconazole, posaconazole, voriconazole, caspofungin and micafungin was performed by a broth microdilution assay according to the methods of the Clinical and Laboratory Standards Institute (CLSI) M38-A2 [6]. Briefly, 100 μl culture preparations (standard RPMI 1640 broth [0.2% dextrose], final inoculum concentrations ranging from 0.4 × 10^4^ to 5 × 10^4^ CFU/ml) were inoculated into 96-well microtiter plates containing 100 μl drug dilutions, and the plates were incubated at 35 °C for 48 h. The minimum inhibitory concentrations (MICs) for amphotericin B, itraconazole, posaconazole, and voriconazole were determined visually as the lowest concentrations with no visible growth (100% inhibition) [6]. For echinocandins (caspofungin and micafungin), the end points were determined as the lowest concentrations of drug leading to the growth of small, rounded, compact hyphal forms in comparison to the hyphal growth observed in the control well (MEC, minimal effective concentration). The quality control strains *Candida krusei* ATCC 6258 and *C. parapsilosis* ATCC 22019 were employed as controls.

A follow-up culture of the wound was performed after 1 month and revealed no fungal growth. Ultimately, we reached a definitive diagnosis of tenosynovitis caused by *S. apiospermum*.

The patient was treated with oral fluconazole 200 mg for 4 weeks. After her discharge from the hospital, we obtained the results of in vitro susceptibility testing of the isolates obtained from the aspirated fluid (Table 1). The last follow-up in the outpatient clinic was 4 months after treatment. The patient recovered completely, with no recurrence.

**Table 1:** The antifungal susceptibility tests

Antifungal agent	100% inhibition MIC (μg/ml)	
	24 h	48 h
Itraconazole	4	>16
Voriconazole	1	1
Posaconazole	2	>16
Fluconazole	1	>64
Amphotericin B	1	4
Micafungin	-	4 (MEC)
Caspofungin	-	4 (MEC)

Cf > *MEC* minimal effective concentration

## Discussion

We report a case of tenosynovitis caused by *S. apiospermum*, which was identified via molecular DNA sequencing. The infectious agent was initially misidentified as an *Alternaria* species via a microscopic examination with lactophenol cotton blue staining. The dematiaceous fungi are defined by melanin or melanin-like pigmentation in the walls of the hyphae and/or spores.

Dematiaceous fungi (melanized fungi), including the *Alternaria* and *Scedosporium* species, cause a wide range of diseases from dermal and epidermal infections in immunocompetent hosts to disseminated sepsis in immunosuppressed hosts [7]. *Alternaria* species can cause human diseases such as cutaneous and subcutaneous infections, sinusitis, ocular infections and, on rare occasions, granulomatous lung disease (especially in immunocompromised hosts). The main clinical manifestation is allergenic respiratory symptoms, which are due to the inhalation of spores. Skin and cutaneous alternariosis mainly occur in immunocompromised hosts [8]. *Alternaria* species are commonly considered to be contaminants. *Alternaria* infections remain locally invasive without any dissemination in most cases. Nonetheless, infections caused by these organisms can be localized, extend to the surrounding tissues (deep extension), or disseminate (hematogenously) to distant organs. Therefore, *Alternaria* dissemination to other tissues should be histologically verified [9]. Fatal *Alternaria* infections were seldomly reported until recently.

However, *Scedosporium* species cause a broad spectrum of clinical diseases such as bronchopulmonary tree infections, localized cutaneous and subcutaneous infections, deep extension or dissemination to distal organs, septic arthritis, osteomyelitis, pneumonia, endocarditis, meningitis and brain abscess [10]. *Scedosporium* has been increasingly reported as a cause of severe infections in immunocompromised hosts; it is known to be pathogenic to both immunocompetent and immunocompromised hosts. Infections due to *Scedosporium* species can result in more than 50% mortality in immunocompromised hosts [3, 4]. Thus, the exact identification of the pathogen is vitally important. The clinical isolate was initially identified as an *Alternaria* species upon a microscopic examination with lactophenol cotton blue staining. However, molecular DNA sequencing revealed that the infection was due to *S. apiospermum*. Tenosynovitis caused by *S. apiospermum* is very rare. Only one case of tenosynovitis (after a dog bite) has been reported world-wide [11]. Previously published data suggest that skin infections due to dematiaceous fungi may be more common in hosts whose activities carry a risk of penetrating trauma. Our patient did not report any recent trauma but lives in a rural area and performed agricultural activities. The definitive diagnosis of a fungal infection relies on the results of clinical mycological cultures and histological findings [12]. One of the studies by Sangoi et al. [13] revealed that fungal pathogens are misclassified in approximately 21% of patients with fungal infections diagnosed histologically but failed to show filamentous fungi when compared with cultures. The histological findings in our case revealed acute and chronic granulomatous inflammation with fungal hyphae and spores, which were stained with Gomori methenamine silver, and the clinical cultures indicated an *Alternaria* species. Through further investigation with DNA sequencing, the infectious agents were identified as *Scedosporium apiospermum* and *Pseudallescheria boydii*.

There is no consensus on the appropriate treatment of dematiaceous fungi infections [14]. Surgical intervention is an important element in the successful treatment of dermal and epidermal infections [15]. In our patient, the treatment consisted of debridement and adjuvant fluconazole therapy based on the mycological culture finding of an *Alternaria* species. There are a few published reports describing successful outcomes with voriconazole for the treatment of *S. apiospermum* in immunosuppressed hosts [16]. Lackner et al. [17] reported low MIC values for voriconazole (MIC 90 with ≤2 ug/ml) in treating *S. apiospermum*. The MIC distribution of other antifungal agents, such as azole and echinocandin, did not reveal any appropriate activity against *Scedosporium* species. The in-vitro susceptibility test with the clinical isolates of our patient showed resistance to fluconazole, itraconazole and posaconazole after 48 h. The infection was susceptible to other antifungals such as voriconazole, amphotericin B, micafungin, and caspofungin. MIC or MEC breakpoints have not been established for *S. apispermum* testing. Therefore, the clinical relevance of testing remains uncertain, and breakpoints with proven relevance have yet to be identified or approved by CLSI or any regulatory agency. As an MIC below 1 μg/ml is usually reported for most molds, including *Aspergillus* species, isolates were grouped as susceptible (MIC or MEC ≤ 1 μg/ml), intermediate (MIC or MEC 2 μg/ml) or resistant (MIC or MEC 4 ≥ μg/ml) for all drugs (CLSI M38). The in vitro susceptibility data are consistent with previous reports. Of the four antifungals tested in this study, voriconazole was found to be the most active against *Scedosporium* species, whereas other antifungals showed limited activity. Skin infections caused by dematiaceous fungi, including *Alternaria* species and *S. apiospermum*, are treated mainly with surgical interventions. *Alternaria* species are susceptible to adjuvant anti-fungal agents, such as fluconazole, but *Scedosporium* species have multidrug-resistant characteristics against many antifungal agents, including azoles and echinocandins [18]. Disseminated infections of *Scedosporium* species. should be treated with antifungal agents. Thus, it is important to distinguish *Alternaria* species from *S. apiospermum*.

In our case with a localized dermal and epidermal infection due to *S. apiospermum* in an immunocompetent patient, irrigation and debridement were more important than anti-fungal treatment.

## Conclusions

This report presents the first case of *S. apiospermum* identified by molecular DNA sequencing, which was initially misidentified as an *Alternaria* species. Physicians should be aware that even though the culture results may indicate an *Alternaria* species infection upon a microscopic examination with lactophenol cotton blue staining, an *S. apiospermum* infection may also be present.

## Abbreviations

C-RP: C-reactive protein
ESR: Erythrocyte sedimentation rate
ITS: Internal transcribed spacer
LPCB: Lactophenol cotton blue
MRI: Magnetic resonance imaging
rDNA: Ribosomal DNA
SDA: Sabouraud’s dextrose agar
WBC: White blood cell
